# The Possible Roles of the Dentate Granule Cell’s Leptin and Other Ciliary Receptors in Alzheimer’s Neuropathology

**DOI:** 10.3390/cells4030253

**Published:** 2015-07-13

**Authors:** James F. Whitfield, Anna Chiarini, Ilaria Dal Prà, Ubaldo Armato, Balu Chakravarthy

**Affiliations:** 1 Human Health Therapeutics, National Research Council of Canada, Ottawa, ON K1A 0R6, Canada; E-Mail: balu.chakravarthy@nrc-cnrc.gc.ca; 2 Histology & Embryology Unit, Department of Life & Reproduction Sciences, University of Verona Medical School, 8 Strada Le Grazie, Verona, Venetia 37134, Italy; E-Mails: anchiari@gmail.com (A.C.); ippdalpra@gmail.com (I.D.P.); uarmato@gmail.com (U.A.)

**Keywords:** adult neurogenesis, Alzheimer’s disease, amyloid-β, amyloid-β oligomers, ciliary LepRb, dentate gyrus, hippocampus, hyperphosphorylated tau, leptin, memory formation, p75^NTR^, somatostatin receptor

## Abstract

Dentate-gyral granule cells in the hippocampus plus dentate gyrus memory-recording/retrieving machine, unlike most other neurons in the brain, are continuously being generated in the adult brain with the important task of separating overlapping patterns of data streaming in from the outside world via the entorhinal cortex. This “adult neurogenesis” is driven by tools in the mature granule cell’s cilium. Here we report our discovery of leptin’s LepRb receptor in this cilium. In addition, we discuss how ciliary LepRb signaling might be involved with ciliary p75^NTR^ and SSTR3 receptors in adult neurogenesis and memory formation as well as attenuation of Alzheimer’s neuropathology by reducing the production of its toxic amyloid-β-derived drivers.

## 1. Introduction

This is a story about a set of extremely important receptor proteins found in the primary cilium protruding from each of the very special little granule cells or neurons of the dentate gyrus, which is part of the ancient hippocampal memory-recording machinery in the brain’s temporal lobes [1]. We are starting to realize now that these receptors are used to build retrievable memories and with them model our outside worlds. One such receptor is LepRb, the functional full-length, signaling receptor of leptin (from the Greek *leptos* meaning thin), a 16-kDa cytokine adipostat, mainly from white fat adipocytes. When carried across the blood-brain-barrier into the hypothalamus, leptin restrains feeding by firing STAT3 (signal transducer and activator of transcription 3), PI3K (phosphoinositide 3-kinase)/Akt (Ak thymoma kinase)/MAPK (mitogen-activated protein kinase)-ERK (extracellular signal-regulated kinase) pathway signals from LepRb into its hypothalamic target cells [2,3,4].

However, we now know that leptin influences many more brain-based functions. The LepRb receptor is expressed in the amygdala, the cerebral cortex, and the hippocampal CA1 field. As comprehensively reviewed by Tezapsidis *et al.* [5] and more recently by Irving and Harvey [6], evidence is mounting that leptin and the cascade of signals from LepRb may arrest Alzheimer’s disease (AD). Indeed convincing evidence supporting leptin-based therapy for AD has been summarized by Neurotez Inc. (Bridgewater, NJ, USA) [7]. However, before going further with the LepRb story, we must first understand the role of dentate gyrus and its ciliated granule cells in memory formation, the developing failure of which is the first detectable sign of on-coming AD.

## 2. The Contribution of the Dentate Gyrus to Memory Formation

**Figure 1:**
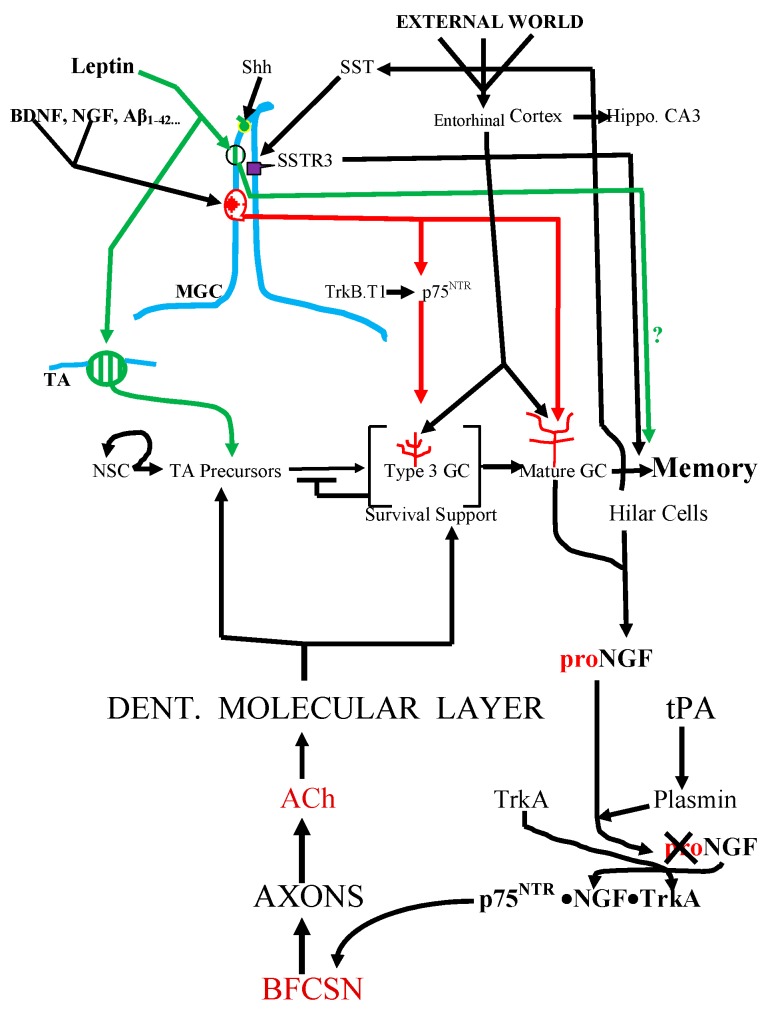
Receptors known to be in the cilium of a dentate granule cell. With the help of ACh (acetylcholine ) from BFCSNs (basal forebrain cholinergic septal neurons), signals from these receptors drive the progression of the progeny of rapidly proliferating initially non-ciliated TA (transit amplifying) cells from the stem cell niche in the dentate gyral SGZ (subgranular zone) to ciliated post-mitotic, mature cells in the overlying granule cell layer. There they receive data from the EC hub and separate overlapping patterns to produce retrievable distinct memories. The “tree-like” structures over the Type 3 and Mature GCs (granule cells) are dendrites. Also note that the green lines and arrows correspond to LepRb functions; red lines and arrows correspond to p75^NTR^ functions; blue line represents primary cilium. Please note the Type 3 GC in this Figure is Immature GC. The symbols: 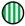 LepRb; 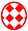 p75^NTR^ 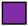 SSTR3.

During a person’s daily activities, the various sensors send streams of data to the different primary sensory regions of the cerebral cortex. Most of these are not retained long enough to be projected into the reverberating *core network* (*i.e.*, the medial and lateral prefrontal cortices, the posterior cingulate and retrosplenial cortices, temporal lobes, and the hippocampus and the associated dentate gyrus) for further processing into a conscious image or “neuronal video” [8]. However, some of the data is sent to the hippocampal formation via the EC (entorhinal cortex) hub where the neurons in layer II process the converging polymodal data for transmission to the memory-encoding machinery in the hippocampal CA3 field and to the dentate gyral granule cells [9,10,11,12,13,14,15,16] (Figure 1). 

However, during a person’s customary daily activities, there is the problem of overlapping data streaming into the hippocampus and dentate gyrus. An efficient memory-recording machine that can fulfill the basic need of a mammal to adapt to a changing environment must be able to separately record even closely similar experiences without producing synaptic palimpsests. Moreover, this machinery must later be able to retrieve these stored component patterns or individual “chunks” and reassemble them into valid though usually experientially modified memories. Therefore, there must be a device that can distinguish overlapping input patterns to enable separate memory storage and retrieval. That device is the dentate gyrus and pattern separation and memory resolution is its job [10,11,12,13,17,18]. In other words, the dentate gyrus prevents palimpsests. 

To do this, the dentate gyrus must be strikingly different from other parts of the brain with their terminally postmitotic neurons [19,20]. Having a permanent population of neurons continuously overwriting past memory circuits would not be the kind of data receiver the owner needs to distinguish between and remember the whats, whens and wheres of similar events. The solution to this problem was to have a constantly renewing pool of hyperexcitable granule cells, each produced initially with “virgin” processes on which to record novel input before maturing, loading up with synapses and finally fading into cellular crones. Adult neurogenesis is what makes the dentate gyrus such a key part of the memory-recording machinery [9,10,11,12,13,14,15,16,17,18,19]. Indeed, only the young adult-born granule cells mediate pattern separation, but as they age, they switch from separating fresh overlapping input patterns to restoring past inputs [21]. However, it should be noted that while a lack of new granule cells impedes the formation of clearly defined memories and distinguishing between overlapping inputs, overloading granule cell networks with new granule cells overwrites and erases pre-existing memory circuits [22].

A lot of effort has been, and is being, spent trying to understand what drives adult neurogenesis and how these adult-born cells contribute to cognition from newborn stem cell daughters to neuronal crones.

## 3. Granule Cells

Adult granule cell production is maintained by a pool of stem cells, known as type 1 radial cells, housed in their special dentate gyral SGZ (subgranular zone) niche [9,19,23,24,25,26]. These type 1 cells have all the features of astrocytes [25]. From their SGZ niche, they shove their apical dendrites through the overlying tightly packed layer of mature granule cells and out into the molecular layer to monitor the activities in the neuronal neighborhood. It is likely that the stem cells and their progeny also form a tightly apposed, multicomponent but intercommunicating SGZ population, the activity within each of its subpopulations being determined by the signals from, and the density of, the contacts with the other subpopulations (Figure 1).

A stem cell resists cell cycle entry to protect its “stemness”, which is the tissue’s “reserve currency”, an excessive expenditure of which may ultimately exhaust the supply of new granule cells. Its decision to start a clone-generating cycle depends on the overlying granule cells’ NMDA receptor activity, a measure of their overall functional adequacy [26,27,28]. A sub-threshold overlying activity will induce the stem cells to start a cycle to make enough functioning mature cells to restore the background activity. An activated stem cell will, on the average, produce one daughter like itself and a second daughter that will keep its cell cycle engine running, leave the niche to produce the first two type 2a TA (transit amplifying) cells, the most proliferatively active of the new clone of progenitor cells [19]. These cells lose their parental radial processes, but they still have stem cell and radial glial cell markers such as glial fibrillary acidic protein and express the *Sox* gene to prevent precocious differentiation. These cells proliferate in clusters around the radial scaffold and capillaries to get growth and development factors such as ADP/ATP, BDNF, and VEGF [9,15,19,23,24,29]. Indeed, this availability of VEGF is why neurogenesis is tightly linked to angiogenesis in the SGZ [26].

The type 2a TA cells generate the less proliferatively active type 2b cells, which by expressing the *NeuroD1* gene shifts from the glial to the neuronal phenotype. These cells are also stimulated by Wnt 3a from surrounding “professional” astrocytes to switch on their *Prox1* gene to produce the specific dentate granule cell marker, which will continue to be needed by their mature progeny to maintain the correct gene expression pattern [9,15]. These type 2b cells are the first of the precursors to express *Tis21*, the product of which will cause their progeny to become terminally post-mitotic by inhibiting cell cycle-initiating cyclin D1 transcription [30]. This *TIS21* expression also marks the switch to full synaptic integration and impending glutamatergic innervations. However, the first synapses formed by the newborns are with axons from GABAergic hilar basket cells, which are not the potent inhibitory response filters for the newborns as they are for the “adult” granule cells. In fact, immature granule cells receive excitatory, instead of inhibitory, signals from GABAergic synapses as they have NKCCl (Na^+^-K^+^-Cl^−^) transporters that hyperpolarize the cells by increasing their intracellular Cl^−^ concentrations. Thus when GABA opens its target Cl^−^ channels, Cl^−^ flows out, depolarizing and exciting the cell. However, when the young type 3 granule cell is maturing, it discards the NKCCl transporter and thus has a low resting Cl^−^ concentration. Therefore, when GABA opens its Cl^−^ channels, Cl^−^ flows into the cell to hyperpolarize and inhibit it [31]. 

“Infant mortality” in the SGZ is normally high. About 50% of the newborns may die without reaching the granule cell layer and, as in most such systems, the signals from the surviving fraction probably feed back to determine the stem cell/TA production level. However, this mortality can be reduced by NGF produced in the dentate gyrus from proNGF by the pro-domain removing tPA (plasminogen activator)-activated plasmin. This “mature”, *i.e.*, “pro-less”, NGF forms p75^NTR^·NGF·TrkA complexes that are carried up the axon and stimulate the distant BFCSNs (basal forebrain cholinergic septal neurons) to make and secrete ACh that promotes newborn granule cell survival [32,33] (Figure 1).

The surviving type 3 progeny of the type 2b cells are migratory neuroblasts. They move up into the lower third of the granule cell layer where they will stay and complete their maturation into functional ciliated granule cells. They must now become part of the dentate gyral ANTs (functionally integrated astrocyte-neuron teams) hill [34]. The incoming granule cells extend their newly formed, “clean-slate” dendrites through the upper granular layer and into the molecular layer and in the process cause the pre-existing astrocytes to reconfigure their structure to form close functional contacts with the arriving dendritic spines and synapses [34]. Now the new granule cells start learning about the outside world as they synapse with the axons from the EC hub (Figure 1).

## 4. Involvement of the Granule Cell’s Primary Cilium in Adult Neurogenesis

We have known since 1963 that mature dentate granule cells have a non-motile, ~4-μm primary cilium [35,36,37,38,39,40]. However, when do they get this antenna? According to Kumamoto *et al.* [41], the early type 2a TA neuroblasts do not have cilia. Indeed that would be expected because the cilium-forming mother centriole and the cytoskeleton are otherwise occupied during rapid cell cycling and any pre-existing cilia are deacetylated and retracted by HDAC6 (histone deacetylase 6). They showed this with specifically marked retroviruses to label the proliferating progenitors and follow their development. Moreover, as expected, they found that the progeny of the proliferating progenitors started producing cilia *only after they had stopped proliferating* (*i.e.*, in stage 3) and were inserting themselves into the mature granule cell layer and pushing their new dendrites through the layer of mature granule cells to meet the glutamatergic axons from the EC (Figure 1). At first sight, this means that preventing cilium formation should prevent migration and maturation but not TA cell proliferation. However, according to Amador-Arjona *et al.* [42] when SGZ cells were deprived of cilia by conditionally turning off their *Ift20* (intraflagellar transport 20 gene [43]), there was a 50% drop in SGZ proliferative activity indicated by a declining fraction of DCX^+^, BrdU^+^, Ki67^+^, and PCNA^+^ cycling cells. Since only TA neuroblasts were affected, does this mean that TA cells are ciliated? Not necessarily, because this reduced proliferation was more likely to increase inhibitory “pressure” on the non-ciliated TA subpopulation from the build-up of deciliated newborns that could not leave the SGZ (Figure 1). Regardless of how the deciliation reduced neurogenesis, it partially impaired novelty recognition and the detection of the escape hole in a Barnes maze [42]. More recently, Berbari *et al.* [36] have also reported that deciliating the murine dentate gyrus and the hippocampal CA1 field by deleting the *IFT88* gene [43] resulted in impaired aversive learning and memory and novel object recognition. Thus, dentate granule cells must have cilia to do their memory recording jobs.

## 5. Looking into the Dentate Granule Cell’s Cilium

It has been known for ~10 years that the murine mature granule cell cilium houses Shh (sonic hedgehog) machinery [44,45] (Figure 1). Indeed Goetz *et al.* [46] have called the primary cilium a “*hedgehog transduction machine*”. In addition, Breunig *et al.* [47] and Han *et al.* [45] have shown that primary cilia regulate adult neurogenesis via Shh signaling.

**Figure 2:**
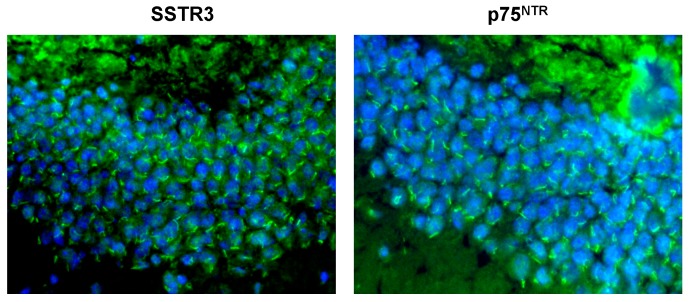
The ciliary localization of p75^NTR^ and SSTR3 in the mature murine dentate granule cells. The identity of the localization of p75^NTR^ with that of cilium-specific SSTR3 establishes p75^NTR^’s ciliary localization. The anti-p75^NTR^ and SSTR3 antibodies and the methodology used to immunostain these cells have been described in [37,38,51].

Then there is the pan-neurotrophin p75^NTR^ receptor in the cilium which can be activated by BDNF, NGF, NT-3, NT4/5, TrkA, TrkB and, as we shall see later, even by the oligomeric Aβ drivers of Alzheimer’s neuropathology [37,38,48,49] (Figure 1 and Figure 2). The localization of p75^NTR^ to the dentate gyral granule cells’ cilia was surprising. It was widely believed that any p75^NTR^ in the dentate gyrus must be in the axons of BFCNs neurons that have been lured to granule cell dendrites in the inner molecular layer to bind to factors such as NGF from granule cells and hilar neurons [32,50,51] (Figure 1). The BFCNs p75^NTR^ would combine with TrkA and NGF (derived from proNGF by TPA-plasmin) to form the p75^NTR^·NGF·TrkA complex. As we said above, this complex stimulates the BFCNs to make and secrete ACh, which promotes the proliferation, survival, and maturation of newborn cells [32,33] (Figure 1).

So one of p75^NTR^’s *non-ciliary* actions is to drive adult neurogenesis. However, what might ciliary p75^NTR^ do? Proliferating (*i.e*., BrdU-labeled) SGZ cells seem to express p75^NTR^ and knocking out the p75^NTR^ gene reduces the proliferating cells and adult neurogenesis by 59%–79% [52,53,54]. However, the proliferatively competent TA cells do not have cilia according to Kumamoto *et al.* [41]. Instead, as we will see in the next paragraph the maturing granule cells’ migration and maturation-stimulating ciliary p75^NTR^ might indirectly determine TA cell proliferation via the SGZ type 3 cell population density and feedback “pressure”. However, any interference with p75^NTR^ gene expression and function would also impede the production by BFCNs of the proliferogenic and survival-promoting ACh (Figure 1).

The most important function of ciliary p75^NTR^ is suggested by the fact that it is the mature granule cells that have the ciliary p75^NTR^ (Figure 2). What could these mature granule cells be doing with their ciliary p75^NTR^? A possible answer to this is suggested by the observation of Kumamoto *et al.* [41] that post-mitotic type 3 newborn granule cells use their cilia to produce dendrites, start migrating upward and connect to the EC axons (Figure 1). *Non-liganded* signals from ciliary p75^NTR^ complexed with those from TrkB T1 (truncated TrkB) could drive the outgrowth of the immature granule cell’s dendritogenic filopodia [55,56,57] (Figure 1). Another participant in arborization might be ceramide, which promotes ciliogenesis and process formation by preventing microtubule deacetylation and retraction via HDAC6 inhibition [58,59]. Ceramide is part of the ring-shaped pericentriolar compartment known as “*the sphingosome*” that is attached to the ciliary basal body where it functions in ciliogenesis and cell migration. Moreover, it has been appropriately shown to promote dendrite arborization in cultured rat embryonic hippocampal neurons [60,61]. The linkage of ceramide to ciliary p75^NTR^ is indicated by the fact that NGF-activated p75^NTR^ stimulates the ceramide-producing membrane-associated sphingomyelinase [62]. Thus, signaling from liganded and non-liganded ciliary p75^NTR^ may indeed drive the ciliogenesis, dendritic arborization, and the exit of newborn cells from the SGZ into the mature granule cell layer indicated by the observations of Kumamoto *et al.* [41]. In addition, the new mature granule cells may retain their ciliary p75^NTR^ to maintain their cilia and modulate their dendritic arbors.

Another receptor in the mature granule cell’s cilium is the non-proliferogenic, GPCR-coupled SSTR3 (somatostatin receptor 3) [37,38,39,40,63,64,65] (Figure 2). Unlike the other four SSTRs (*i.e.*, SSTRs 1, 2, 4, and 5), SSTR3 is confined to primary cilia by a specific cilium-targeting amino acid sequence in its third intracellular loop, *i.e.*, the I3-CTS [63]. Therefore, it is important for what follows in the next section that if a cell produces something that co-localizes to a protruding ~4-μm structure along with SSTR3, *that protruding structure is a cilium and that something must also be ciliary.*

Stanić *et al.* [40] have found that *newborn* neuroblasts do not put SSTR3 into their cilia, but the postmitotic mature granule cells in the granule cell layer, such as those in Figure 2, have loaded it into their cilia at a later stage of their development. Obviously, they put it into their cilia to do a “mature” job related to cognition. That job is memory encoding because without it, mice cannot recognize novel objects [64]. In addition, a lack of SSTR3 could be at least partially responsible for the cognitive deficit caused by granule cell deciliation recently reported by Berbari *et al.* [63].

Since adult-born granule cells do not have cilia until the type 3 stage [41], we will suggest below that a SSTR3-like delayed cilium loading may also apply to LepRb (Figure 1).

## 6. The Ciliary Leptin Receptor in Adult Neurogenesis and AD

As we said in the *Introduction*, there is evidence for an involvement of leptin and its functional LepRb receptor in memory formation and possibly reducing the core drivers of Alzheimer’s disease (AD), a very important possibility for our growing population of seniors.

The common (>95%) form of AD, LOAD (*i.e.*, late onset AD) or Sporadic AD starts its initially unnoticed, decades-long journey through the brain from its “Ground Zero” in the lateral EC [66]. From there it spreads through existing neural circuitry, leaving disconnected networks, an atrophic hippocampus, and eventually a noticeably declining cognition in its wake [67].

**Figure 3:**
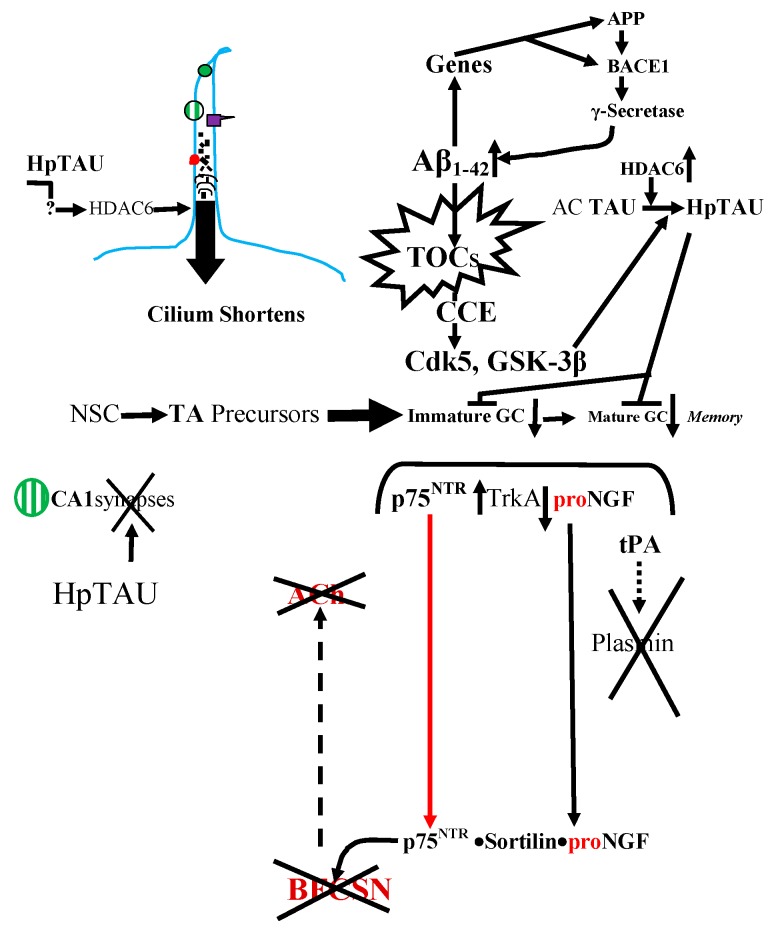
Some possible interactions between the dentate gyral granule cells’ ciliary receptors, AβOs (toxic oligomeric Aβ_1–42_) and toxic hyperphosphorylated TAU (Hp-TAU in the Figure and pTAU in the text) that together drive the spread of Alzheimer’s neuropathology from the EC to interconnected brain regions. As illustrated here, in the core of AD pathology are accumulating Aβ_42_ monomers that aggregate into configurationally dynamic toxic oligomeric “cocktails” (TOCs), which produce a cascade of protein kinases that generate the similarly self-inducing toxic pTAU by several ways. At first AC-TAU (acetylated TAU) resists hyperphosphorylation and aggregation, but the level of HDAC6 (histone deacetylase 6) rises in the AD brain and deacetylates TAU, which can then be hyperphosphorylated. One of the ways pTAU is made is by triggering CCE (cell cycle re-entry) of supposed terminally post-mitotic neurons that cannot enter prophase but still activate Cdk1-cyclin B1 mitotic kinase, eject Cdk5 from their nuclei into the cytoplasm, and activate the key cytoplasmic GSK-3β where they produce the toxic pTAU instead of initiating prophase. As explained in the text, the newborn granule cells no longer have the survival support of ACh and they also face the self-inducing toxic pTAU produced by the similarly self-inducing AβOs. The reduced neurogenesis in the slowly atrophying hippocampal structure signals stem cells to generate TA cells to replace lost cells but this is a futile effort because of the high mortality of their progeny. In addition, pTAU causes granule cells’ cilia to shrink, which might reduce the arborization, maturation, and migration-driving signaling from the p75^NTR^ receptor. Note: pTAU is Hp-TAU in the figure to emphasize the *hyper*phosphorylation of the peptide. NSC: Neural stem cells. The green lines and arrows correspond to LepRb functions; red lines and arrows correspond to p75^NTR^ functions; blue line represents primary cilium. The symbols: 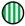 LepRb; 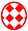 p75^NTR^ 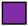 SSTR3.

The accumulating primary drivers of the slowly advancing pathology perhaps decades before the appearance of symptoms seem to be AβOs (Aβ_42_ peptide oligomers) [68,69,70] (Figure 3). Active neurons in healthy young brains produce physiologically functioning and non-toxic Aβ_42_ monomers via the sequential actions of BACE1 (beta-site amyloid precursor protein-cleaving enzyme 1) and γ-secretases that chop the Aβ_42_ off APP (amyloid-β precursor protein) (Figure 3). The resulting pieces are packaged into vesicles, transported down axons to their terminals, from which they are released during synaptic activity [71,72]. In the young brain, the cellular levels of the Aβ_42_ monomers and its variously processed and potentially dangerous derivatives are kept at a safe picomolar level by several proteases, such as neprilysin, as well as by ejection from the brain by LRP1 (low density lipoprotein receptor-related protein 1), the potent Aβ_42_ efflux driver [73]. However, with advancing age, declining disposal mechanisms enable the levels of Aβ_1–42_ peptides and their toxic derivatives and configurations to climb into the danger zone and start making “cocktails” of dynamically toxic AβOs, the TOCs (toxic oligomeric “cocktails”) in Figure 3 [68,70,74]. One of the reasons for the excessive buildup of the AβOs-forming Aβ_42_ is the loss, early in the spread of AD pathology, of the production of the neprilysin-promoting SST (somatostatin) by SST-producing cells in the dentate gyral hilus and the hippocampal CA1-CA3 fields [75,76] (Figure 3).

**Figure 4:**
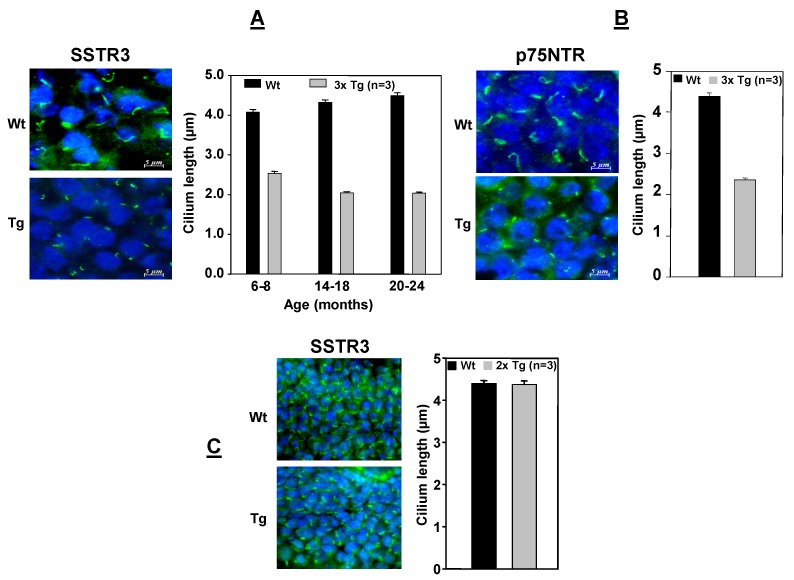
A ciliary example of the toxic Aβ_42_/pTAU “two-step” of Ittner and Götz [85]. (**A**): Accumulating Aβ_1–42_ alone in 2x AD-transgenic mice does not affect the length of granule cilia. (**B**) and (**C**): But the length of granule cells’ cilia is shorter in 3x AD-transgenic mice accumulating Aβ_1–42_ and expressing a human mutant Tau when immunostained with SSTR3 and p75^NTR^ antibodies. It is likely, as shown in Figure 3, that the shortening is due to pTAU-promoted tubulin deacetylation and the resulting fragmentation of the microtubular axoneme by HDAC6 [58,87,88]. The experimental details may be found in Chakravarthy *et al.* [37].

Another consequence of the Aβ_42_ buildup is an increased expression of the p75^NTR^ pan-neurotrophin receptor as demonstrated in both cultured SH-SY5Y human neuroblasts and hippocampal membranes from AD patients [37,38,51,77]. However, it is unlikely that the mature dentate granule cells’ ciliary p75^NTR^ contributes to this rise and its consequences because we have observed that these cilia are significantly shortened (e.g., from 4- to 2.2-μm; *p* < 0.001) in 3xTg AD mice [37] (Figure 3 and Figure 4). However, increased non-ciliary p75^NTR^ in other hippocampal fields, such as CA1, and its stimulation by Aβ_42_ [48,49] combines with drops in TrkA and tPA to form a toxic p75^NTR^·Sortilin·proNGF complex instead of the normal 75^NTR^·NGF·TrkA complex [32,78] (Figure 1 and Figure 3). The p75^NTR^·Sortilin·proNGF complex kills BFCSN cells and with them their adult neurogenesis-promoting ACh secretion [32] (Figure 3).

Since adult neurogenesis normally drops with age in humans and rodents [13], it presumably should drop faster with the surging AβOs. Nevertheless, the increased mortality of newborn cells might feed-back and stimulate NSC and the non-ciliated TA cells (Figure 3). Moreover, there are indeed reports of increased proliferation in the early AD stages [79,80,81]. However, despite this, neurogenesis drops because the newborn neurons neither survive nor mature. This happens in part because of the action of pTAU, the other toxic and self-inducing AD driver, and in part owing to the lack of ACh due to the destruction of BFCSNs by the p75^NTR^·Sortilin·proNGF complex (Figure 3). Moreover, over time the drop in neurogenesis and declining mature cell population, perhaps because of excessive, apoptogenic ciliary p75^NTR^ activation, reflexively activates stem and TA cell proliferation and consequently depletes the stem cell pool [79,81] (Figure 3).

Aβ_42_ is a triply dangerous peptide. Besides forming the toxic AβOs, it is a *self*-*inducing pathology* “*seed*” and a *transcription factor*! Either exogenous or endogenous Aβ_42_ peptides can enter the nuclei of both astrocytes and neurons and promote their own endogenous production by activating the genes for the APP precursor and the BACE1 protease [82,83] (Figure 3). This increases Aβ_42_/AβOs secretion and spreading, reciprocal recruitment of the astrocyte-neuron members of the brain’s ANTs [84]. But as we said above, AβOs are not alone. As Ittner and Götz [85] have said, Aβ_42_ and p-TAU form a toxic “*pas de deux*” (two-step) or, to over-paraphrase Bloom [86], Aβ_42_ is the AD gun and hyperphosphorylated TAUes are its bullets.

The granule cell cilia provide an example of the toxic collaboration of the two AD drivers. These cilia are unaffected in 2xTg-AD mice which only accumulate Aβ_42_ (Figure 4), but they are significantly shorter in 3xTg-AD mice which accumulate Aβ_42_ and express human mutant Tau (Figure 4) [37]. A clue to the cause of this Tau-dependant shortening is the reduced tubulin acetylation and significantly elevated HDAC6 deacetylase in the cortices and hippocampi of human AD brains [87]. This cilial shortening may be due to HDAC6 deacetylating and thereby fragmenting the ciliary axonemal microtubules [58,87,88] (Figure 3).

One-way AβO might induce the AD gun is to induce extrasynaptic NMDA receptors to let Ca^2+^ surge into the neurons. This activates AMPK, CAMKII and GSK-3β, which phosphorylate several Tau residues [69,89] (Figure 3). This reconfigures Tau to prevent it from working with kinesin motors to carry cargo to the axon terminus [89,90,91]. Instead of staying in the axon pTAU ectopically redistributes to the soma, dendrites, and synapses [86,92]. pTAU becomes a potent network disruptor by forming a complex with the *Fyn* tyrosine kinase. The AβOs start this by binding to the normal prion protein (PrP^C^) in the postsynaptic plasma membrane [90]. This activates *Fyn*, which phosphorylates Tau tyrosine residues and binds to pTAU. The resulting *Fyn*·pTAU complex then invades dendritic spines where it targets and phosphorylates NMDA receptors and induces them to admit a flood of Ca^2+^ that destroys synapses and with them the postsynaptic machinery [86,92,93] (Figure 3). With this, the toxic pTAU is released in exosomes and spreads the neuropathology to its contacts [93].

However, there is another way to hyperphosphorylate Tau, which we have chosen to illustrate in Figure 3. Even before a person has reached the MCI (mild cognitive impairment) stage of the disease, AβOs have caused a large number of supposedly terminally post-mitotic neurons to overexpress MiRNA-26b. This RNA species downregulates the Rb (retinoblastoma) protein that is part of a cell cycle genes-associated complex consisting of Rb·E2F·Cdk5, which has been preventing CCE (cell cycle re-entry) by blocking the expression of major cell cycle proteins [94]. MiRNA-26b over-expression and Rb downregulation result in the dispersal of the genes-blocking complex, which starts a series of events leading to DNA replication. Indeed, the cell may finish replicating its chromosomes but it cannot initiate mitosis [94,95]. Nevertheless, it still expresses the Cdk1-cyclin B1 mitotic kinase, which in a normal cell would be activated and promptly sent into the nucleus to trigger nuclear membrane breakdown and chromosome condensation. However, in a CCE neuron it *stays in the cytoplasm*, where it can access and phosphorylate Tau [95]. However, much before this, the MiRNA-26b-triggered dispersal of the nuclear CCE-blocking Rb·E2F·Cdk5 complex releases the Cdk5 kinase which enters the cytoplasm where it can also access and phosphorylate Tau and phosphorylate and inactivate any available Rb [94].

Thus, infectious AβOs have various ways to the produce their pTAU “*bullets*” and with them spread AD pathology. The messages from all of this are clear: AβOs are at the heart of AD pathology and to attenuate AD pathology you must stop the production of TOCs and their induction of pTAU bullets.

## 7. Leptin Reduces Aβs Production

**Figure 5:**
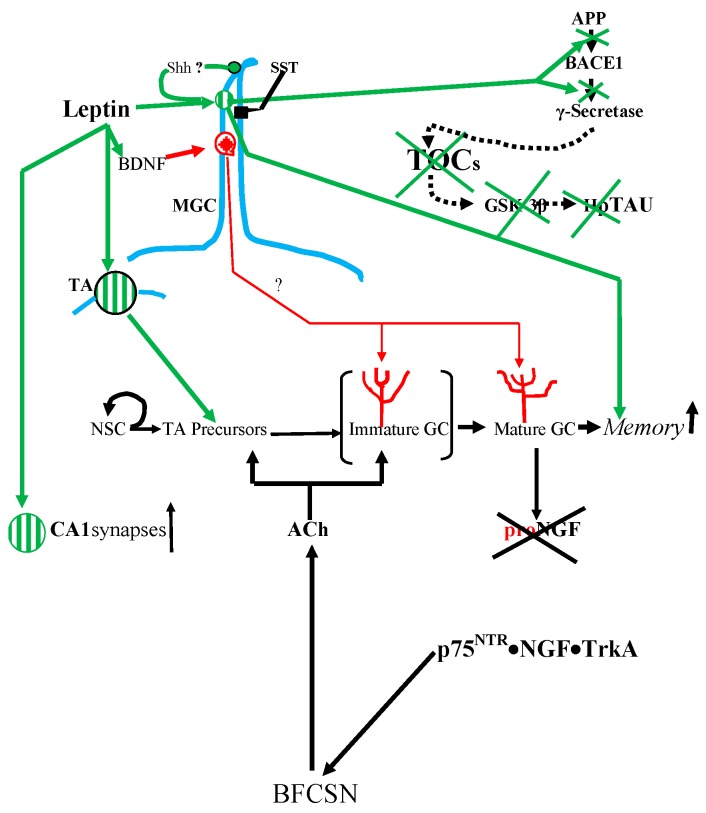
This figure, based on the mechanisms discussed in the text, describes how leptin-induced signaling by preciliary and ciliary LepRb in dentate granule cells and non-ciliary synaptic LepRb in hippocampal CA1 neurons might reduce the AD-induced loss of memory by attenuating or preventing the production of the toxic TOCs and pTAU in Figure 3. The green lines and arrows correspond to LepRb functions; red lines and arrows correspond to p75^NTR^ functions; blue line represents primary cilium. The symbols: 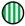 LepRb; 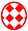 p75^NTR^ 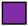 SSTR3.

It seems likely from experiments on AD-model transgenic mice as well as rats that leptin/LepRb might also be able to hit the source of the AD drivers in humans [6,96,97,98]. As we have seen, the primary drivers of AD pathology most likely are the AβOs. As shown in Figure 3, Aβ_42_, the non-toxic source of the toxic AβOs, is cut out of the APP precursor by BACE1 and γ-secretase. Leptin strikes at AD pathology by reducing Aβ_1–42_ production via a SIRT1 (histone deacetylase)-mediated reduction of BACE1 expression [99] (Figure 5). Moreover, besides reducing AβOs, leptin decreases the production of the synapse-destroying pTAU by inhibiting a principal Tau kinase, GSK-3β [96] (Figure 5). By reducing Aβ_1–42_ production, leptin also prevents the destructive surging of caspase 3 in synapses and thus the decline in synaptic density in the CA1 field and the associated hippocampus-related memory deficits [100] (Figure 5). However, what does leptin do to adult neurogenesis? Garza *et al.* [101] found that 14 days of intermittent intraperitoneal injections of leptin (1.0 mg/kg) into adult C5BL/6J mice significantly stimulated proliferation in the animals’ SGZs without affecting differentiation or survival of the newborn cells (Figure 1). Leptin also stimulated the STAT3/PI3k/Akt-mediated proliferation of cultured adult dentate progenitor cells. They later reported that intraperitoneal leptin injections into male Sprague-Dawley rats also overcame the glucocorticoid inhibition of adult neurogenesis possibly by stimulating BDNF expression [102].

Obviously, it would have been less traumatic than intraperitoneal injections to continuously and endogenously administer leptin. In addition, Pérez-González *et al.* [103] were able to do this elegantly. They produced a stable, elevated intracerebral level of leptin by injecting a HIV-Leptin gene-carrying lentiviral vector into the lateral cerebral ventricles of APP/PS1 AD-model transgenic mice. During the following 3 months, the implanted, virally vectored leptin gene activity had stimulated dentate gyral progenitor proliferation and attenuated Aβs-induced neurodegeneration.

## 8. Where Do Dentate Granule Cells Put their LepRb?

Because leptin stimulates adult neurogenesis by increasing progenitor proliferation but not maturation, we suspected that it might have been working through cilia, which, with their Shh machinery, have been shown to be involved in adult neurogenesis [42,43,44,45,47,104].

**Figure 6:**
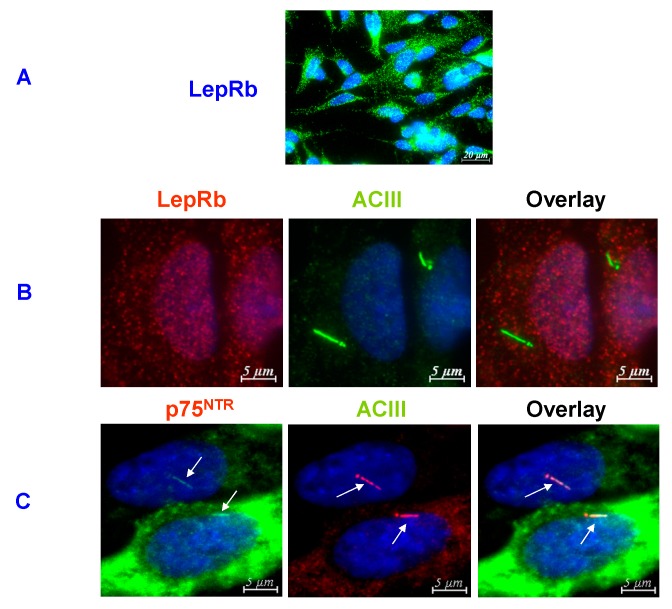
(**A**). An example of the immunostainable non-ciliary punctate distribution of LepRb in human SH-SY5Y neuroblastoma cells that is like the non-ciliary punctate LepRb distributions in cultured rat CA1 and cultured murine dentate granule cells [101,105]. (**B**). These neuroblastoma cells do not have the cilium-specific SSTR3, but they have the cilium-specific ACIII (adenylyl cyclase III). Here we see that the immunostained Lep Rb particulates are not ciliary, *i.e.*, are not co-localized with ACIII. It should be noted that Greco *et al.* [108] have shown, and we have confirmed (data not shown), that these neuroblasts are leptin responsive. C. It should also be noted that the cilia of the non-cycling SH-SY5Y cells in these cultures did contain p75^NTR^ (immunostained as described in [37,38,51]), which would be consistent with the ability of the p75^NTR^-induced ceramide production to drive cilium formation [58,59,60,61]. The cells were cultured as described by Chakravarthy *et al.* [109] and the anti-LepRb monoclonal IgG_1_ antibody (Ob-R, B-3, and sc8391) was purchased from Santa Cruz Biotechnology Inc. (CA). Polyclonal anti-ACIII antibody (C-20, sc-588) was also purchased from Santa Cruz Biotechnology.

As comprehensively reviewed in [6], Harvey *et al.* have focused on leptin/LepRb’s ability to target activities in the hippocampal CA1 field. However, CA1 is not an adult neurogenesis site. Nevertheless, they cultured rat CA1 pyramidal neurons and found LepRb punctately distributed in cytoplasm, plasma membrane, and most importantly in synapses, but *not in cilia* (see Figure 6 of Shanley *et al.* [105]). Leptin stimulates transmission at CA1 synapses and induces LTP in slices of adult CA1 tissue by increasing AMPA receptor trafficking and synaptic strength. Garza *et al.* [101], on the other hand, focused on the murine dentate gyrus and its SGZ. They, like Shanley *et al.* [105], immunostained cultured neurons for LepRb (their Figure 4B). Here too LepRb was *not ciliary*. They also found the same immunostainable punctuate cytoplasmic and perinuclear LepRb granules as did Shanley *et al.* [105]. Indeed, we have found the same distribution of immunostainable LepRb granules in cultured, leptin-responsive SH-SY5Y human neuroblasts (Figure 6). As can be seen, these human neuroblasts were ciliated and at least some had even put p75^NTR^ into their cilia like the mature murine dentate granule cells, but again LepRb was still *not ciliary*. [106,107]

**Figure 7:**
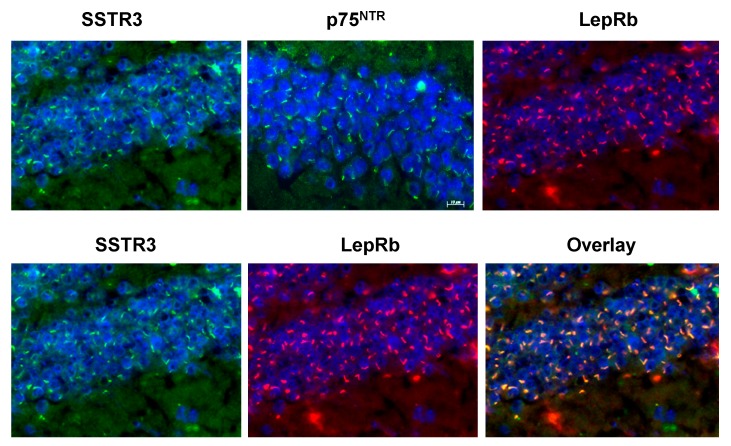
LepRb is detectably localized only to the primary cilia of mature murine dentate granule cells along with p75^NTR^ and SSTR3 receptors. Hemi-brains from 7–8 months-old female wild-type C57BL6 mice were immunostained with LepRb, p75^NTR^, or SSTR3 antibody as described in Figure 2 and Figure 6 and in [37,38,51]. Similar results were obtained with three 14–18 months-old female mice and one male mouse. When co-immunostained with LepRb monoclonal antibody and either SSTR3 or p75^NTR^ polyclonal antibody, the signals from the LepRb and SSTR3 or p75^NTR^ (data not shown) antibody merged (overlay) indicating ciliary co-localization. In all immunohistochemical studies, Dako fluorescent mounting medium containing DAPI DNA-binding nuclear stain was used and the tissue sections were examined with an Olympus fluorescence microscope as described in [37,38]. A similar demonstration of ciliary LepRB will also appear in Whitfield *et al.* [110].

From these examples, it would seem that LepRb is *not ciliary*. However, in these cases the localization may have been affected by cellular age and other factors. In our hands, using the same C57/BL6 strain of mice from the same place (Jackson Laboratories, Bar Harbor, Me) as Garza *et al.* [101] the *in vivo* mature granule cells seemingly confined LepR to their cilia (Figure 7). There were no clusters of immunostainable granules like those that we found in the proliferating SH-SY5Y neuroblasts (Figure 6). As shown in Figure 7, the granule cells’ LepRb-containing “protrusions” also contained p75^NTR^, which we have proven to be confined to mature granule cell cilia [37,38]. Even more convincing, they also contained SSTR3, which specifically binds only to cilia with its APSQ ciliary localization amino acid sequence [63].

How might this differently localized LepRb affect the leptin-induced signaling pathway in cultured and *in vivo* neurons? According to Garza *et al.* [102], the LepRb signal in the cultured granule cells follows the JAK2/STAT3/PI3K/Akt pathway as it does in the other cultured neurons. However, does this apply to the ciliary LepRb’s signal pathway, especially since the cilium is loaded with the proliferogenic Shh signal transduction mechanism needed for adult neurogenesis [45,47] (Figure 1)? How might these pathways be linked in the SGZ newborns? A clue is provided by the incompatibility of cell cycling and ciliation. As expected, the rapidly proliferating TA cells do not have cilia [41]. Instead, it is their non-proliferating, type 3 progeny that produce cilia when they start extruding processes and migrating upward into the mature granule cell layer. So it is likely that at this early stage the LepRB is still in the cell membrane, from which leptin stimulates their proliferation (Figure 1 and Figure 4). Thus, when these cells are cultured they would only have the punctate distribution of pre-ciliary LeptRb reported by Garza *et al.* [101] and in the leptin-stimulable (data not shown) SH-SY5Y neuroblasts in Figure 6.

This also suggests that the dentate granule cells only load LepRb into their cilia after they start maturing. This is similar to the later ciliary loading of SSTR3 reported by Stanić *et al*. [40]. Thus ciliary LepRb may also be involved in a mature granule cell function such as memory encoding like SSTR3 [36,64] Figure 1.

## 9. Conclusions

From the aforesaid it follows that recording information about the outside world streaming in from the EC hub and preventing any partially overlapping data from palimpsesting on recipient networks is the job of the very special, adult-born dentate granule cell (at least in mice). Such cells are equipped with ciliary receptor proteinsincluding leptin’s LepRb receptor, the pan-neurotrophin p75^NTR^ receptor and the SSTR3 receptor. As summarized in Figure 1, it appears that newborn TA neurons may first use non-ciliary LepRb signaling to stimulate their proliferation. Then the TA cells’ progeny use their ciliary p75^NTR^ to drive the production of CA3 field-connecting mossy axons and EC-connecting dendrites and migration from the SGZ to the mature dentate granule cell layer. Then as they settle in to their final “home”, they load SSTR3 and LepRb into their cilia to start recording memories.

These are exciting questions for future studies. However, these are really just some possible functions of dentate granule cell’s cilium. There is likely to be more than the three potent receptor/drivers that we have been discussing. We have a long way to go to understand how the granule cells use their ciliary tools to drive adult neurogenesis, encode memories and attenuate AD-like pathology in transgenic mice. Most importantly, it is important to find out whether LepRb signaling is an AD therapeutic or more likely an AD preventative for humans. It is unlikely that signals from dispersed or ciliary LepRb can repair damage already inflicted by toxic AβO “cocktails” and Hp-Tau probably because this damage is irreversible (Figure 5). However, the continuous provision of leptin by a cerebrally implanted leptin gene did prevent further damage in AD-model transgenic mice [98]. In addition, it probably did so by blocking the productions of Aβ_1–42_, the source of the self-inducing toxic AβOs and Hp-Taues (Figure 5). Thus, leptin’s anti-amyloidogenic activity is the basis of Neurotez’s patent [7] for treating AD with leptin and its derivatives. 

Finally, if leptin really can target AβOs accumulation in humans as well as rodents, providing a steady supply of leptin may at least slow the progression of AD pathology in early-stage patients. Perhaps its effectiveness could also be increased with agents such as the CaSR (Ca^2+^-sensing receptor)-antagonizing calcilytic drug NPS 2143 or a similarly acting one that stops the oversecretion of endogenous Aβs from both astrocytes and neurones of the human cerebral cortex [84,106] and the ~5-kDa ABP (Amyloid-β peptide) that selectively binds AβOs [107].

## References

[1] Duvernoy H., Cattin F., Risold P.Y. (2013). The Human Hippocampus.

[2] Ladyman S.R., Grattan D.R. (2013). JAK-STAT and feeding. JAKSTAT.

[3] Levin B.E., Magnan C., Dunn-Meynell A., Le Foll C. (2011). Metabolic sensing in the brain: Who, what, where and how?. Endocrinology.

[4] Li M.-D. (2011). Leptin and beyond: An odyssey to the central control of body weight. Yale J. Biol. Med..

[5] Tezapsidis N., Johnston J.M., Smith M.A., Ashford J.W., Casadesus G., Robakis N.K., Wo-lozin B., Perry G., Zhu X., Greco S.J. (2009). Leptin: A novel therapeutic strategy for Alz-heimer’s disease. J. Alzheimers Dis..

[6] Irving A.J., Harvey J. (2013). Leptin regulation of hippocampal synaptic function in health and disease. Philos. Trans. R. Soc. Lond. B Biol. Sci..

[7] Tezapsidis N. (2012). Leptin as an anti-amyloidogenic biologic and methods for delaying the onset and reducing Alzheimer’s disease-like pathology. US Patent.

[8] Dehaene S. (2014). Consciousness and the Brain: Deciphering How the Brain Decodes Our Thoughts.

[9] Aimone J.B., Li Y., Lee S.W., Clemenson G.D., Deng W., Gage F.H. (2014). Regulation and function of adult neurogenesis: From genes to cognition. Physiol. Rev..

[10] Cameron H.A., Glover L.R. (2015). Adult neurogenesis: Beyond learning and memory. Annu. Rev. Psychol..

[11] Drew L.J., Fusi S., Hen R. (2013). Adult neurogenesis in the mammalian hippocampus: Why the dentate gyrus?. Learn. Mem..

[12] Hunsaker M.R., Kesner R.P. (2013). The operation of pattern separation and pattern completion processes associated with attributes or domains of memory. Neurosci. Behav. Rev..

[13] Seib D.R.H., Martin-Villalba A. (2014). Neurogenesis in the normal ageing hippocampus: A mini review. Gerontology.

[14] Witter M.P. (2007). Intrinsic and extrinsic wiring of CA3: Indications for connectional heterogeneity. Learn. Mem..

[15] Yu D.X., Marchetto M.C., Gage F.H. (2014). How to make a hippocampal dentate gyrus granule neuron. Development.

[16] Benarroch E.E. (2013). Adult neurogenesis in the dentate gyrus. Neurology.

[17] Moscovitch M., Rosenbaum R.S., Gilboa A., Addis D.R., Westmacott R., Grady C., McAndrews M.P., Levine B., Black S.M., Winocur G. (2005). Functional neuroanatomy of remote episodic, semantic and spatial memory: A unified account. J. Anat..

[18] Rugg M.D., Johnson J.D., Park H., Uncapher M.R. (2008). Encoding-retrieval overlap in human episodic memory: A functional neuroimaging perspective. Prog. Brain Res..

[19] Kempermann G. (2011). Adult Neurogenesis 2.

[20] Treves A., Tashiro A., Witter M.E., Moser E.I. (2008). What is the dentate gyrus good for?. Neuroscience.

[21] Nikashiba T., Cushman J.D., Pelkey K.A., Renauddineau S., Buhl D.L., McHugh T.J., Barrera V., Chittajallu R., Iwamoto K.S., McBain C.J. (2012). Young dentate granule cells mediate pattern separation, whereas old granule cells facilitate pattern completion. Cell.

[22] Mongiat L.A., Schinder A.F. (2014). Neuroscience: A price to pay for adult neurogenesis. Science.

[23] Basak O., Taylor V. (2009). Stem cells of the adult mammalian brain and their niche. Cell Mol. Life Sci..

[24] Goldman S.A., Chen Z. (2011). Perivascular instruction of cell genesis and fate in the adult brain. Nat. Neurosci..

[25] Morrens J., van den Broeck W., Kempermann G. (2012). Glial cells in adult neurogenesis. Glia.

[26] Suh H., Deng W., Gage F.H. (2009). Signaling in adult neurogenesis. Annu. Rev. Cell Dev. Biol..

[27] Gould E., Cameron H.A. (1996). Regulation of neuronal birth, migration, and death in the rat dentate gyrus. Dev. Neurosci..

[28] Nacher J., Rosell D.R., Alonso-Liosa G., McEwen B.S. (2001). NMDA receptor antagonist treatment induces a long-lasting increase in the number of proliferating cells, PSA-NCAM-immunoreactive granule neurons and radial glia in the adult rat dentate gyrus. Eur. J. Neurosci..

[29] Antequara D., Portero A., Bolos M., Orive G., Hernández R.M., Pedraz J.L.K., Carro E. (2012). Encapsulated VEGF-secreting cells enhance proliferation of neuronal progenitors in the hippocampus of AβPP/Ps1 mice. J. Alzheimers Dis..

[30] Attardo A., Fabel K., Krebs J., Haubensak W., Huttner W.B., Kempermann G. (2010). Tis21 expression marks not only populations of neurogenic precursor cells but also new post-mitotic neurons in adult hippocampal neurogenesis. Cereb. Cortex.

[31] Mejia-Gervacio S., Murray K., Lledoi P.-M. (2011). NKCCl controls GABAergic signaling and neuroblast migration in the postnatal forebrain. Neural Dev..

[32] Bruel-Jungerman E., Lucassen P.L., Francis F. (2011). Cholinergic influences on cortical development and adult neurogenesis. Behav. Brain Res..

[33] Van der Borght K., Mulder J., Keijser J., Eggen B.J., Luiten P.G., van der Zee E.A. (2005). Input from the medial septum regulates adult hippocampal neurogenesis. Brain Res. Bull..

[34] Krzisch M., Temprana S.G., Mongiat L.A., Armida J., Schmutz V., Virtanen M.A., Kocher-Braissant J., Kraftsik R., Vutskits L., Conzelmann K.K. (2014). Pre-existing astrocytes form functional processes on neurons generated in the adult hippocampus. Brain Struct. Funct..

[35] Dahl H.A. (1963). Fine structure of cilia in the rat cerebral cortex. Z. Zellforsch. Mikrosk. Anat..

[36] Berbari N.F., Malarkey E.B., Zaki S.M., Yazdi S.M., McNair A.D., Kippe J.M., Croyle M.J., Kraft T.W., Yoder B.K. (2014). Hippocampal and cortical primary cilia are required for aversive memory in mice. PLoS ONE.

[37] Chakravarthy B., Gaudet C., Ménard M., Brown L., Atkinson T., Laferla F.M., Ito S., Armato U., dal Prà I., Whitfield J. (2012). Reduction of the immunostainable length of the hppocampal dentate granule cells’ primary cilia in 3 x AD-transgenic mice producing human Aβ(1–42) and tau. Biochem. Biophys. Res. Commun..

[38] Chakravarthy B., Gaudet C., Ménard M., Atkinson T., Chiarini A., dal Prà I., Whit-Field J. (2010). The p75 neurotrophin receptor is localized to primary cilia in adult mouse hippocampal dentate gyrus granule cells. Biochem. Biophys. Res. Commun..

[39] Händel M., Schultz S., Stenarius A., Schreff M., Erdtmann-Vouriliotis M., Schmidt H., Wolf G., Höllt V. (1999). Selective targeting of somatostatin receptor 3 to neuronal cilia. Neuroscience.

[40] Stanić D., Malmgren H., He H., Scott L., Aperia A., Hökfelt T. (2009). Developmental changes in frequency of the somatostatin receptor 3 protein. Brain Res..

[41] Kumamoto N., Gu Y., Wang J., Jamoschka S., Takemaru K.-L., Levine J., Ge S. (2012). A role for primary cilia in glutamatergic synaptic integration of adult-born neurons. Nat. Neurosci..

[42] Amador-Arjona A., Eelliott J., Miller A., Ginbey A., Pazour G.J., Enikolopov G., Roberts A.J., Terskikh A.V. (2011). Primary cilia regulate proliferation of amplifying progenitors in adult hippocampus: Implications for learning and memory. J. Neurosci..

[43] Malicki J., Avidor-Reiss T. (2014). From cytoplasm into the cilium: Bon voyage. Organogenesis.

[44] Corbit K.C., Aanstad P., Singla V., Norman A.R., Stainier D.Y., Reiter J.F. (2005). Verteb-rate smoothened functions at the primary cilium. Nature.

[45] Han Y.G., Spassky N., Romaguera-Ros M., Garcia-Verdugo J.M., Aguilar A., Schneider-Maunoury S., Alvarez-Buylla A. (2008). Hedgehog signaling and primary cilia are required for formation of adult neural stem cells. Nat. Neurosci..

[46] Goetz S.C., Ocbina P.J.R., Anderson K.V. (2009). The primary cilium as a hedgehog signal transduction machine. Methods Cell Biol..

[47] Breunig J.J., Sarkisian M.R., Arellano J.I., Morozov Y.M., Ayoub A.E., So-jitra S., Wang B., Flavell R.A., Rakic P., Town T. (2008). Primary cilia regulate hippo-campal neurogenesis by mediating sonic hedgehog signaling. Proc. Natl. Acad. Sci. USA.

[48] Chiarini A., dal Prà I., Whitfield J.F., Armato U. (2006). The killing of neurons by beta-amyloid peptides, prions, and proinflammatory cytokines. Ital. J. Anat. Embryol..

[49] Sotthibundhu A., Li Q.X., Thangnipon W., Coulson E.J. (2009). Abeta(1–42) stimulates adult SVZ neurogenesis through the p75 neurotrophin receptor. Neurobiol. Aging.

[50] Makuch R., Baratta J., Karaelias L.D., Lauterborn J.C., Gall C.M., Yu J., Robertson R.T. (2001). Arrival of afferents and the differentiation of target neurons: Studies of developing cholinergic projections to the dentate gyrus. Neuroscience.

[51] Chakravarthy B., Ménard M., Ito S., Gaudet C., dal Prà I., Armato U., Whit-Field J.F. (2012). Hippocampal membrane-associated p75^NTR^ levels are increased in Alzheimer’s disease. J. Alzheimers Dis..

[52] Bernabeu R.O., Longo F.M. (2010). The p75 neurotrophin receptor is expressed by adult mouse dentate progenitor cells and regulates neuronal and non-neuronal cell genesis. BMC Neurosci..

[53] Catts V.S., Al-Menhali N., Burne T.H., Colditz M.J., Coulson E.J. (2010). The p75 neuro-trophin receptor regulates hippocampal neurogenesis and related behaviours. Eur. J. Neurosci..

[54] Colditz M.J., Catts V.S., Al-Menhali N., Osborne G.W., Bartlett P.F., Coulson E.J. (2010). p75 neurotrophin receptor regulates basal and fluoxetine-stimulated hippocampal neurogenesis. Exp. Brain Res..

[55] Hartmann M., Brigadski T., Erdmann K.S., Holtmann B., Sendtner M., Narz F., Lessmann V. (2004). Truncated TrkB receptor-induced outgrowth of dendritic filopodia involves the p75 neurotrophin receptor. J. Cell Sci..

[56] Klein R., Conway D., Parada L.F., Barbacid M. (1990). The trkB tyrosine protein kinase gene codes for a second neurogenic receptor that lacks the catalytic kinase domain. Cell.

[57] Yacoubian T.A., Lo D.C. (2000). Truncated and full-length trkB receptors regulate distinct modes of dendritic growth. Nat. Neurosci..

[58] He Q., Wang G., Wakade S., Dasgupta S., Dinkins M., Kong J.N., Spassieva S.D., Bieberich E. (2014). Primary cilia in stem cells and neural progenitors are regulated by neutral sphingomyelinase 2 and ceramide. Mol. Biol. Cell.

[59] Wang G., Krishnamurthy K., Bieberich E. (2009). Regulation of primary cilia formation by ceramide. J. Lipid Res..

[60] Brann A.B., Scott R., Neuberger Y., Abulafia D., Boldin S., Fainzilber M., Futerman A.H. (1999). Ceramide signaling downstream of the p75 neurotrophin receptor mediates the effects of nerve growth factor on outgrowth of cultured hippocampal neurons. J. Neurosci..

[61] Schwarz A., Futerman A.H. (1998). Inhibition of sphingolipid synthesis, but not degradation, alters the rate of dendritic growth in cultured hippocampal neurons. Brain Res. Dev. Brain Res..

[62] Dobrowski R.T., Carter B.D. (1998). Coupling of the p75 neurotrophin receptor to sphingo-lipid signaling. Ann. N. Y. Acad. Sci..

[63] Berbari N.F., Johnson A.D., Lewis J.S., Askwith C.C., Mykytyn K. (2008). Identification ciliary localization sequences within the third intracellular loop of G protein-coupled receptors. Mol. Biol. Cell.

[64] Einstein E.B., Patterson C.A., Hon B.J., Regan K.A., Reddi J., Melnikoff D.E., Mateer M.J., Schultz S., Johnson B.N., Tallent M.K. (2010). Somatostatin signaling in neuronal cilia is critical for object recognition memory. J. Neurosci..

[65] Green J.A., Gu C., Mykytyn K. (2012). Heteromerization of ciliary G protein-coupled receptors in the mouse brain. PLOS ONE.

[66] Khan U.A., Liu L., Provenzano F.A., Berman D.E., Profaci C.P., Sloan R., Mayeux R., Duff K.E., Small S.A. (2014). Molecular drivers and cortical spread of lateral entorhinal cortex dysfunction in preclinical Alzheimer’s disease. Nat. Neurosci..

[67] Penzes P., Vanleeuwen J.E. (2011). Impaired regulation of synaptic actin cytoskeleton in Alzheimer’s disease. Brain Res..

[68] Hubin E., van Nuland N.A., Broersen K., Pauwels K. (2014). Transient dynamics of Aβ contribute to toxicity in Alzheimer’s disease. Cell. Mol. Life Sci..

[69] Klein W.L. (2013). Synaptotoxic amyloid-β oligomers: A molecular basis for the cause, diag-nosis and treatment of Alzheimer’s disease?. J. Alzheimers Dis..

[70] Selkoe D.J., Mandelkow E., Holtzman D.M. (2012). The Biology of Alzheimer Disease.

[71] Cheng X., Wu J., Geng M., Xiong J. (2014). The role of synaptic activity in the regulation of amyloid beta levels in Alzheimer’s disease. Neurobiol. Aging.

[72] Choy R.W., Cheng Z., Schekman R. (2012). Amyloid precursor protein (APP) traffic from the cell surface via endosomes for amyloid β production in the trans-Golgi network. Proc. Natl. Acad. Sci. USA.

[73] Zlokovic B.V. (2011). Neurovascular pathways to neurodegeneration in Alzheimer’s disease and other disorders. Nat. Rev. Neurosci..

[74] Jawhar S., Wirths O., Bayer T.A. (2011). Pyroglutamate amyloid-β (Aβ): A hatchet man in Alzheimer disease. J. Biol. Chem..

[75] Burgos-Ramos E., Hervás-Aguilar A., Aguado-Liera D., Puebla-Jiménez L., Hernández-Pinto A.M., Barrios V., Arilla-Ferreiro E. (2008). Somatostatin and Alzheimer’s disease. Mol. Cell. Endocrinol..

[76] Ramos B., Baglietto-Vargas D., del Rio J.C., Moreno-Gonzalez I., Santa-Maria C., Jimenez S., Caballero C., Lopez-Tellez J.F., Khan Z.U., Ruano D. (2006). Early neuropathology of somatostatin/NPY GABAergic cells in the hippocampus of a PSI × APP transgenic model of Alzheimer’s disease. Neurobiol. Aging.

[77] Ito S., Ménard M., Atkinson T., Gaudet C., Brown L., Whitfield J., Chakravarthy B. (2012). Involvement of insulin-like growth factor 1 receptor signaling in the amyloid-beta peptide oligomers-induced p75 neurotrophin receptor protein expression in mouse hippocampus. J. Alzheimers Dis..

[78] Bruno A.M., Leon W., Fragosos G., Mushynski W., Almazan G., Cuello A.C. (2009). Amyloid beta-induced nerve growth factor dysmetabolism in Alzheimer’s disease. J. Neuropathol. Exp. Neurol..

[79] Shruster A., Melamed E., Offen D. (2010). Neurogenesis in the aged and neurodegenerative brain. Apoptosis.

[80] Waldau R., Shetty A.K. (2008). Behavior of neural stem cells in the Alzheimer brain. Cell. Mol. Life Sci..

[81] Yu Y., He J., Zhang Y., Luo H., Zhu S., Yang Y., Zhao T., Wu J., Huang Y., Kong J. (2009). Increased hippocampal neurogenesis in the progressive stage of Alzheimer’s disease phenotype in an APP/PS1 double transgenic mouse model. Hippocampus.

[82] Barucker C., Harmeier A., Weiske J., Fauler B., Albring K.F., Prokop S., Hildebrand P., Lurz R., Heppner F.L., Huber O. (2014). Nuclear trans-location uncovers the amyloid peptide Aβ_42_ as a regulator of gene transcription. J. Biol. Chem..

[83] Maloney B., Lahiri D.K. (2011). The Alzheimer’s amyloid β-peptide (Aβ) binds a specific DNA Aβ-interacting domain (AβID) in the APP, BACE1, and APOE promoters in a sequence-specific manner: Characterizing a new regulatory motif. Gene.

[84] Dal Prà I., Chiarini A., Gui L., Chakravarthy B., Pacchiana R., Gardenal E., Whitfield J.F., Armato U. (2015). Do astrocytes collaborate with neurons in spreading the “infectious” Aβ and Tau drivers of Alzheimer’s disease?. Neuroscientist.

[85] Ittner L.M., Götz J. (2011). Amyloid-β and tau—A toxic *pas de deux* in Alzheimer’s disease. Nat. Rev. Neurosci..

[86] Bloom G.S. (2014). Amyloid-β and tau: The trigger and bullet in Alzheimer disease patho-genesis. JAMA Neurol..

[87] Ding H., Dolan P.J., Johnson G.V.W. (2008). Histone deacetylase 6 interacts with the micro-tubule-associated protein tau. J. Neurochem..

[88] Xiong Y., Zhao K., Wu J., Xu Z., Jin S., Zhang Y.Q. HDAC6 mutations rescue human tauinduced induced microtubule defects in *Drosophila*. Proc. Natl. Acad. Sci. USA.

[89] Seward M.E., Swanson E., Norambuena A., Reimann A., Cochran J.N., Li R., Robertson E.D., Bloom G.S. (2013). Amyloid-β signals through tau to drive ectopic neuronal cell cycle reentry in Alzheimer’s disease. J. Cell Sci..

[90] Larson M., Sherman M.A., Amar F., Nuvolone M., Schneider J.A., Bennett D.A., Aguzzi A., Lesnć S.E. (2012). The complex PrP(c) Fyn couples human oligomeric Aβ with pathological tau changes in Alzheimer’s disease. J. Neurosci..

[91] Ward S.M., Himmelstein D.S., Lancia J.K., Binder L.I. (2012). Tau oligomers and tau toxicity in neurodegenerative disease. Biochem. Soc. Trans..

[92] Nisbet R.M., Polanco J.-C., Ittner L.M., Götz J. (2015). Tau aggregation and its interplay amyloid-β. Acta Neuropathol..

[93] Spires-Jones T.L., Hyman B.T. (2014). The intersection of amyloid beta and tau at synapses in Alzheimer’s disease. Neuron.

[94] Absalon S., Kochanek D.M., Raghavan V., Krichevsky A.M. (2013). MiR-26b, upregulated in Alzheimer’s disease, activates cell cycle entry tau phosphorylation, and apoptosis in postmitotic neurons. J. Neurosci..

[95] Hussman J.W., Nochlin D., Vincent J. (2000). Mitotic activation: A convergent mechanism for a cohort of neurodegenerative diseases. Neurobiol. Aging.

[96] Greco S.J., Sarkar S., Casadesus G., Zhu X., Smith M.A., Ashford J.W., Johston J.M., Tezapsidis N. (2009). Leptin inhibits glycogen synthase kinase-3β to prevent tau phosphorylation in neuronal cells. Neurosci. Lett..

[97] Marwarha G., Ghribi O. (2012). Leptin signaling and Alzheimer’s disease. Am. J. Neurodegener. Dis..

[98] McGregor G., Malekizadeh Y., Harvey J. (2015). Minireview: Food for thought: Regulation of synaptic function by metabolic hormones. Mol. Endocrinol..

[99] Marwarha G., Raza S., Meiers C., Ghribi O. (2014). Leptin attenuates BACE1 expression and amyloid-β genesis via the activation of SIRT1 signaling pathway. Biochim. Biophys Acta.

[100] Pérez-González R., Alvira-Botero M.X., Robayo O., Antequera D., Garzón M., Martin-Moreno A.M., Brera B., de Ceballos M.I., Carro E. (2014). Leptin gene therapy attenuates neural damages evoked by amyloid-β and reduces memory deficits in APP/PS1 mice. Gene Ther..

[101] Garza J.C., Guo M., Zhang W., Lu X.-Y. (2008). Leptin increases adult hippocampal neurogenesis *in vivo* and *in vitro*. J. Biol. Chem..

[102] Garza J.C., Guo M., Zhang W., Lu X.-Y. (2012). Leptin restores adult hippo-campal neurogenesis suppressed by chronic unpredictable stress and reverses glucocorticoid-induced inhibition of GSK3β/β-catenin signaling. Mol. Psychiatry.

[103] Pérez-González R., Antequera D., Vargas T., Spuch C., Bolós M., Carro E. (2011). Leptin induces proliferation of neuronal progenitors and neuroprotection in a mouse model of Alzheimer’s disease. J. Alzheimers Dis..

[104] Kenney A.M., Rowitch D.H. (2000). Sonic hedgehog promotes G_1_ cyclin expression expression and sustained cell cycle progression in mammalian neuronal precursors. Mol. Cell. Biol..

[105] Shanley L.J., O’Malley D., Irving A.J., Ashford M.L., Harvey J. (2002). Leptin inhibits epileptiform-like activity in rat hippocampal neurones via PI3-kinase-driven activation of BK channels. J. Physiol..

[106] Armato U., Chiarini A., Chakravarthy B., Chioffi F., Pacchiana R., Colarusso E., Whitfield J.F., dal Prà I. (2013). Calcium-sensing receptor antagonist (calcilytic) (NPS2143 specifically blocks the the increased secretion of endogenous fibrillary or soluble Aβ_25–35_ in human cortical astrocytes and neurons—Therapeutic relevance to Alzheimer’s disease. Biochim. Biophys. Acta.

[107] Chakravarthy B., Ménard M., Brown L., Hewitt M., Atkinson T., Whitfield J.F. (2013). A synthetic peptide corresponding to a region of the pericentriolar material 1 (PCM-1) protein binds β amyloid (Aβ_1–42_) oligomers. J. Neurochem..

[108] Greco S.J., Sarkar S., Johnston J.M., Zhu X., Su B., Casadesus G., Ashford J.W., Smith M.A., Tezapsidis N. (2008). Leptin reduces Alzheimer’s disease-related tau phosphorylation in neuronal cells. Biochem. Biophys. Res. Commun..

[109] Chakravarthy B., Gaudet C., Ménard M., Atkinson T., Brown L., Laferla F.M., Armato U., Whitfield J.F. (2010). Amyloid-beta peptides stuimulate the expression of the p75(NTR) neurotrophin receptor in SHSY5Y human neuroblastoma cells and AD transgenic mice. J. Alzheimers Dis..

[110] Whitfield J.F., Chakravarthy B., Chiarini A., Dal Prá I., Armato U., Costa A., Villalba E. (2015). The leptin receptor, a driver of adult neurogenesis that may treat Alzheimer’s disease, has been found in murine dentate gran-ule cells’ ciliary toolbox. Horizons in Neuroscience Research.

